# Association between prolonged corticosteroids use in COVID-19 and increased mortality in hospitalized patients: a retrospective study with inverse probability of treatment weighting analysis

**DOI:** 10.1186/s13054-023-04434-5

**Published:** 2023-04-15

**Authors:** Marina Verçoza Viana, José Augusto Santos Pellegrini, Amanda Vilaverde Perez, Patrícia Schwarz, Daiandy da Silva, Cassiano Teixeira, Marcelo Basso Gazzana, Tatiana Helena Rech

**Affiliations:** 1 Intensive Care Unit, Hospital de Clínicas de Porto Alegre, Ramiro Barcelos 2350, Porto Alegre, RS 90035-003 Brazil; 2 Graduate Program in Medicine: Medical Sciences, Universidade Federal do Rio Grande do Sul, Porto Alegre, RS Brazil; 3 Graduate Program in Medical Sciences: Endocrinology, Universidade Federal do Rio Grande do Sul, Porto Alegre, RS Brazil; 4 Clinical Pharmacy Unit, Hospital de Clínicas de Porto Alegre, Porto Alegre, RS Brazil; 5 Graduate Program in Pneumology, Universidade Federal do Rio Grande do Sul, Porto Alegre, RS Brazil; 6 Pneumology Division, Hospital de Clínicas de Porto Alegre, Porto Alegre, RS Brazil; 7 Internal Medicine and Rehabilitation Sciences, Universidade Federal de Ciências da Saúde de Porto Alegre, Porto Alegre, Brazil

**Keywords:** SARS-CoV-2, Corticosteroids, Critical illness, Mortality, Inverse probability of treatment weighting

## Abstract

**Background:**

Previous studies have demonstrated a beneficial effect of early use of corticosteroids in patients with COVID-19. This study aimed to compare hospitalized patients with COVID-19 who received short-course corticosteroid treatment with those who received prolonged-course corticosteroid treatment to determine whether prolonged use of corticosteroids improves clinical outcomes, including mortality.

**Methods:**

This is a retrospective cohort study including adult patients with positive testing for Sars-CoV-2 hospitalized for more than 10 days. Data were obtained from electronic medical records. Patients were divided into two groups, according to the duration of treatment with corticosteroids: a short-course (10 days) and a prolonged-course (longer than 10 days) group. Inverse probability treatment weighting (IPTW) analysis was used to evaluate whether prolonged use of corticosteroids improved outcomes. The primary outcome was in-hospital mortality. Secondary outcomes were hospital infection and the association of different doses of corticosteroids with hospital mortality. Restricted cubic splines were used to assess the nonlinear association between mortality and dose and duration of corticosteroids use.

**Results:**

We enrolled 1,539 patients with COVID-19. Among them, 1127 received corticosteroids for more than 10 days (prolonged-course group). The in-hospital mortality was higher in patients that received prolonged course corticosteroids (39.5% vs. 26%, p < 0.001). The IPTW revealed that prolonged use of corticosteroids significantly increased mortality [relative risk (RR) = 1.52, 95% confidence interval (95% CI): 1.24–1.89]. In comparison to short course treatment, the cubic spline analysis showed an inverted U-shaped curve for mortality, with the highest risk associated with the prolonged use at 30 days (RR = 1.50, 95% CI 1.21–1.78).

**Conclusions:**

Prolonged course of treatment with corticosteroids in hospitalized patients with COVID-19 was associated with higher mortality.

**Supplementary Information:**

The online version contains supplementary material available at 10.1186/s13054-023-04434-5.

## Background

The dysregulated inflammatory and immune response seen in viral pneumonia can lead to the development of acute lung injury [1]. Corticosteroids are commonly used to reduce this response and improve outcomes [2]. The use of corticosteroids in patients with acute respiratory distress syndrome (ARDS) has been studied for decades [3] with recommendations for early use, before fibrosis develops [4]. In patients with COVID-19, early use of dexamethasone has been shown to reduce mortality in those requiring respiratory support [5]. Additionally, a meta-analysis of seven studies demonstrated that systemic corticosteroids, including dexamethasone, hydrocortisone, and methylprednisolone, reduced 28-day mortality in critically ill patients with COVID-19 [1].

While the benefits of corticosteroids in the acute phase of COVID-19 are clear, the optimal duration of treatment is less well established. Previous studies suggested that early use of corticosteroids may be more effective in reducing mortality and mitigating the development of fibrosis [1]. However, it is still unclear if prolonged use of corticosteroids in patients with COVID-19 provides any additional clinical benefits or if it results in any adverse events.

A retrospective study by Mongardon et al. found no difference in mortality or duration of mechanical ventilation in patients who received corticosteroids late in the course of COVID-19-induced ARDS compared to those who did not receive corticosteroids [6]. However, the study may have been underpowered to detect a benefit of late use of corticosteroids. Therefore, this study aims to compare patients who received short-course corticosteroids treatment for COVID-19 with patients who received prolonged-course corticosteroid treatment. By evaluating these two groups, we seek to determine if the prolonged use of corticosteroids improves clinical outcomes, including mortality.

## Methods

### Study design and participants

This is a retrospective cohort study. Patients were considered eligible if they were > 18 years old and were admitted to the Hospital de Clínicas de Porto Alegre, Brazil, from May 2020 to June 2021 with positive testing for SARS-CoV-2. Patients without treatment with corticosteroids, less than 10 days of hospital stay, or death before 10 days of hospital admission (not eligible for prolonged corticosteroids use) were excluded as criteria. Readmissions were also excluded. Time from admission to initiation of steroids was not an inclusion nor an exclusion criterion for study entry.

Patients were divided into two groups: one group received corticosteroids for 10 days (short-course group) and the other group received corticosteroids for longer than 10 days (prolonged-course group). All patients that received corticosteroids for longer than 10 days were classified as prolonged-course, regardless of when the treatment with corticosteroids were started (early or late in the course of the disease). The cut-off value of 10 days was chosen for prolonged courses based on recent guidelines [1]. Patients admitted at the intensive care unit (ICU) and received mechanical ventilation (MV) in the first 48 h of hospital admission were defined as critically ill. Others were classified as non-critically ill patients (ward patients). The decision and time to initiate corticosteroid therapy was a responsibility of the attending physicians. The corticosteroids most prescribed at our institution are prednisone, hydrocortisone, methylprednisolone, and dexamethasone.

All procedures were in accordance with the ethical standards of the National Research Committee and with the 1964 Declaration of Helsinki. This study was approved by the Research Ethics Committee of the Hospital de Clínicas de Porto Alegre, Brazil (2021-0220). Informed consent was waived by the institution for reasons of the retrospective design and anonymization of patient identifiers before analysis.

### Procedures

The diagnosis of COVID-19 infection was based on results from polymerase chain reaction (PCR) testing for SARS-CoV-2 using nasal swabs. No asymptomatic patients or patients with mild disease were included. All included patients presented at least moderate disease, defined as those who experience symptoms such as shortness of breath, chest pain, and hemoptysis, requiring hospitalization for oxygen therapy, but not admitted to the ICU. Patients with severe disease were those with respiratory distress, significant decrease in blood oxygen levels, and signs of organ failure, requiring hospitalization and eventually mechanical ventilation. Critically ill patients were those with the most severe types of COVID-19, characterized by respiratory failure, shock, and multi-organ dysfunction [7]. Patients with severe disease and critically ill patients were analyzed separately.

Clinical and laboratory data were obtained for all patients via electronic medical records. The variables included age, sex, C-reactive protein (CRP), D-dimer, and lactate dehydrogenase (LDH) levels, presence of positive blood cultures, number of different antibiotics used, days under antibiotics treatment, need for early ICU admission (defined as ICU admission < 48 h), need for ICU admission at any time, need for mechanical ventilation, time spent on MV, ICU and hospital length of stay, and mortality. CRP levels were quantified using turbidimetric immunoassay with normal values < 5 mg/L. D-dimer levels were quantified by latex agglutination assay and considered normal if < 500 ng/mL. LDH levels were quantified by enzymatic assay with normal values < 220 U/L. Type, dose, and duration of corticosteroids used were obtained for all patients. Methylprednisolone-equivalent dosing was used for comparison [8]. Accordingly, 1 mg of dexamethasone was considered equivalent to 5.33 mg of methylprednisolone and 1 mg of hydrocortisone was equivalent to 0.2 mg of methylprednisolone. The maximum dose of methylprednisolone equivalent received in a single dose at any time during hospitalization was recorded.

### Outcomes

The primary outcome was in-hospital mortality. The secondary outcomes were hospital infection defined by positive cultures, antibiotics needed, days under antibiotics treatment, and the association of different doses of corticosteroids with hospital mortality. For critically ill patients, the following outcomes were evaluated: ICU length of stay, time on MV, and ICU mortality.

### Statistical analysis

No observational or randomized trial addressed our specific research question. Thus, the RECOVERY trial was used to determine the appropriate sample size, since our comparison group underwent treatment for the same time as the intervention group in the RECOVERY [4]. Considering that patients who received prolonged corticosteroid present a benefit for mortality similar to that described for patients under MV in the RECOVERY trial (23.3% vs 26.2%), the required sample size was calculated to be 2189 patients, using the G*Power software program (version 3.1) to detect a 3% absolute difference between the groups, with 80% power and a 5% alpha error.

Data were presented as mean ± standard deviation, median (interquartile range [IQR]), or number (percentage). Comparisons between groups were performed using one-way analysis of variance, the Mann–Whitney test, or the Fisher’s test, as appropriate. Missing data were not imputed.

Inverse probability of treatment weighting (IPTW) was used to allow causal inference in this observational data [9]. The propensity score model was calculated using the full matching method for receiving prolonged dose corticosteroids. The following covariates were included in the model: age, need for ICU admission < 48 h, sex, hospital length of stay, and CRP levels. These predictors were chosen based on clinical judgment and model fit. Afterwards, Poisson regression was performed weighted with the propensity score. No missing data was imputed.

To mitigate the substantial loss of information resulting from the categorization of corticosteroid duration and dose, an analysis utilizing restricted cubic splines was employed to examine the nonlinear relationship between mortality and both the dose and duration of corticosteroid use. Restricted cubic splines have the capacity to identify local features and generate dependable estimates at the extremities of data, rendering the spline model suitable for detecting marginal treatment effects [10]. The knots were defined by quantiles 0.10, 0.50, 0.90 [11]. Regarding the duration of corticosteroids, these quantiles corresponded to eight, 15, and 37 days and for the dose of corticosteroids they corresponded to 32, 46, and 125 mg of methylprednisolone equivalent dose.

A Poisson regression analysis was conducted to examine the association between mortality and time duration in days, adjusting for age, sex, need for ICU admission < 48 h, and CRP levels. Relative risk (RR) was calculated for each time point in comparison to 10 days. Additionally, an analysis was conducted to compare the equivalent of methylprednisolone with each dose point, using a 32 mg dose of methylprednisolone as the reference point. The overall association between methylprednisolone equivalent dose and duration of corticosteroids in days with mortality was assessed using the Wald test.

Statistical analyses were performed using the R software program (version 4.2.2, The R Foundation for Scientific Computing) and differences were considered statistically significant at p values < 0.05.

## Results

### Study population

**Fig. 1:**
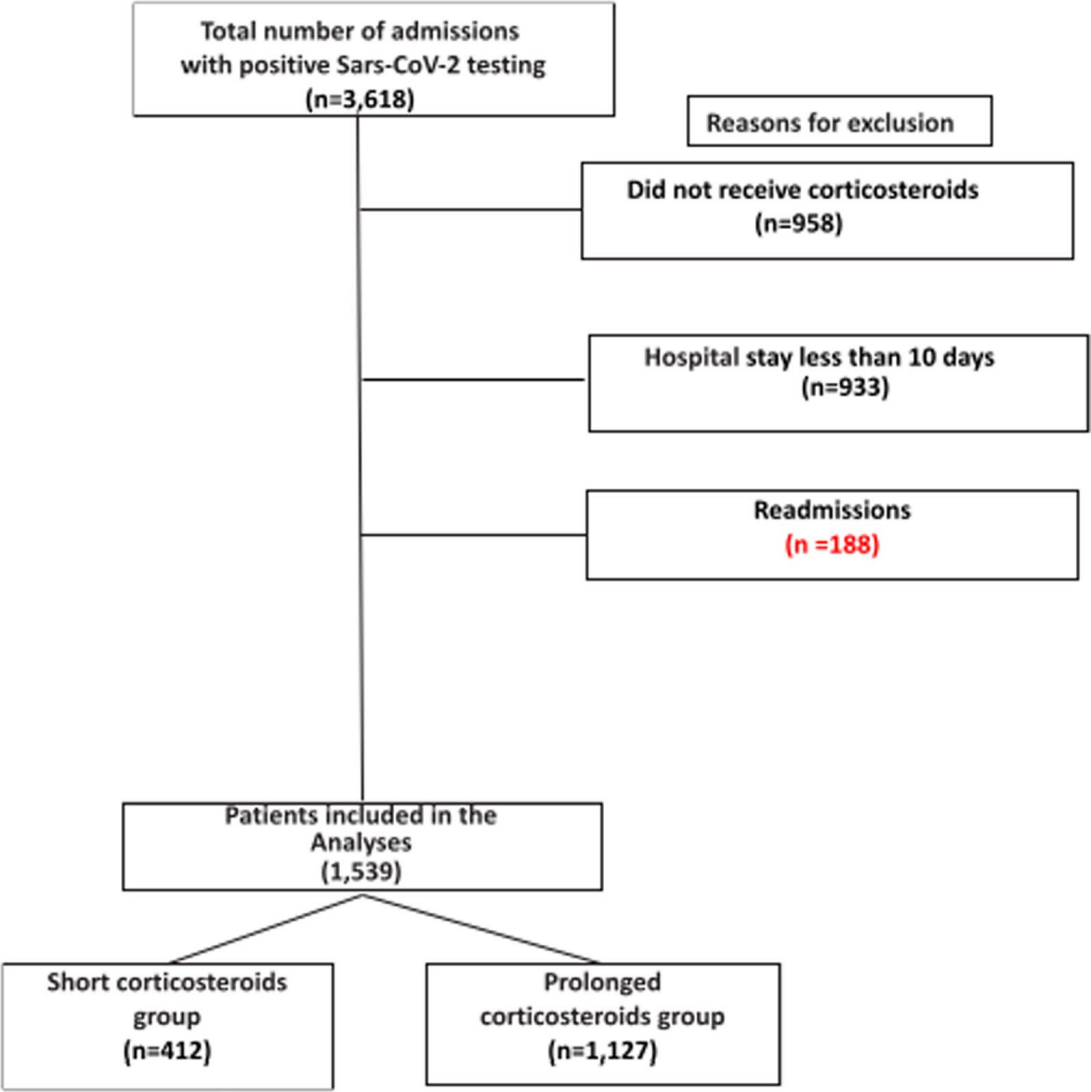
Patient eligibility and enrollment. Patients in the short group received corticosteroids only at the first 10 days of hospital admission. Patients in the prolonged group received corticosteroids at the first 10 days and further. Patients not exposed to the possible use of prolonged corticosteroids were excluded

In total, 3,618 patients were admitted to the hospital with positive testing for Sars-CoV-2 from May 2020 to June 2021. From then, 1,539 were analyzed. Figure 1 describes patient eligibility and reasons for exclusions.

Table 1 summarizes the main baseline characteristics of the study population. A total of 412 (26.8%) patients received short-course corticosteroids and 1127 (73.2%) received prolonged-course corticosteroids. Most patients were male (54.4%, n = 837) and the mean age was 59 ± 15 years. Non-critically ill (ward) patients totalized 917 individuals (59.6%) and 622 (40.4%) were critically ill (ICU) patients.

**Table 1:** Baseline characteristics of patients

Characteristic	Overall (n = 1,539)	Short corticosteroids (n = 412)	Prolonged corticosteroids (n = 1,127)	p
All hospitalized patients (n = 1539)				
Demographics				
Age (years)	59 ± 15	61 ± 15	59 ± 14	0.008
Men (n, %)	837 (54.4)	221 (53.6)	616 (54.7)	0.766
Need for ICU at any time (n, %)	1,364 (88.6)	338 (82)	1,026 (91)	< 0.001
Use of corticosteroids				
Maximum MED (mg)	46 (32–80)	32 (32–36)	56 (36–96)	< 0.001
Total length of corticosteroids use (days)	15 (10–24)	9 (7–10)	19 (14–28)	< 0.001
Biochemical measurements				
CRP (mg/dL)	121 (62–199)	114 (56–188)	125 (67–206)	0.014
LDH (U/L)	481 (337–642)	455 (311–599)	491 (339–663)	0.024
D-dimer (mg/L)	1.9 (0.9–5.5)	1.9 (0.9–5.3)	1.9 (0.9–5.6)	0.869

	Overall (n = 622)	Short corticosteroids (n = 163)	Prolonged corticosteroids (n = 459)	p
Critically ill patients (n = 622)				
Demographic				
Age (years old)	57 ± 14	57 ± 15	57 ± 14	0.748
Men (n, %)	347 (55.8)	90 (55.2)	257 (56)	0.936
Use of corticosteroids				
Maximum MED (mg)	53 (2.5–90)	32 (32–40)	64 (40–100)	< 0.001
Total duration of corticosteroid use (days)	15 (10–26)	9 (7–10)	20 (14–30)	< 0.001
Time from hospital admission to initiation of corticosteroids (days)	0 (0–0)	0 (0–1)	0 (0–0)	< 0.001
Biochemical measurements				
CRP (mg/dL)	139 (83–219)	127 (75–197)	145 (84–229)	0.066
LDH (U/L)	553 (415–729)	527 (450–647)	562 (412–757)	0.315
D-dimer (mg/L)	2.3 (1.1–8.7)	2.3 (1.2–10.4)	2.3 (1.1–7.6)	0.376

Values are mean ± SD or median and interquartile range

*ICU* intensive care unit, *MV* mechanical ventilation, *MED* methylprednisolone equivalent dose, *CRP* C-reactive protein, *LDH* lactate dehydrogenase

The average time from hospital admission to initiation of corticosteroid therapy was zero days for the overall population of the study (Table 1).

### In-hospital mortality and clinical outcomes in the overall population

**Fig. 2:**
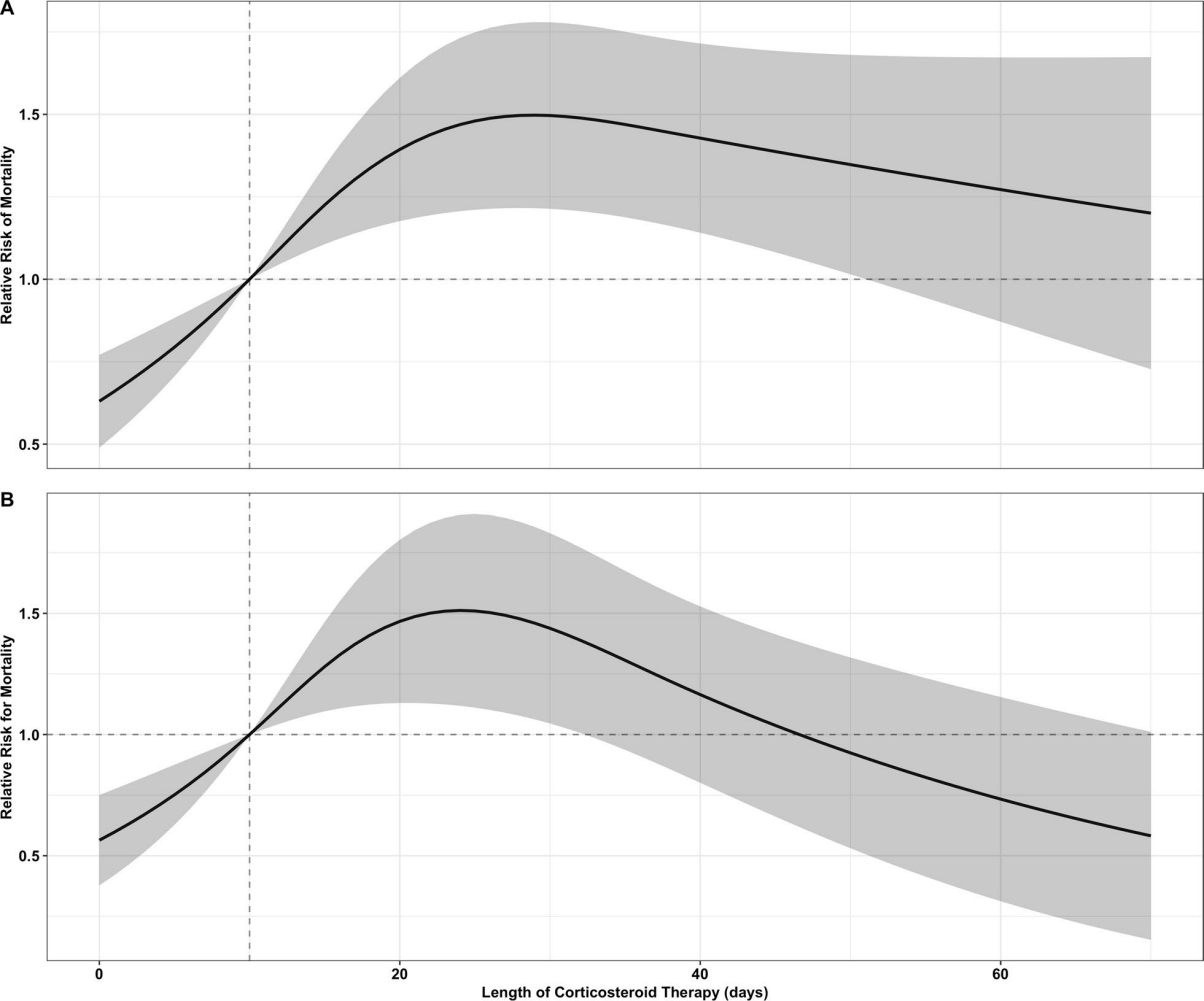
Non-linear association between duration of corticosteroids use and mortality. Non-linear association between duration of corticosteroids and mortality in hospitalized (panel **A**) and critically ill (panel **B**) patients using restricted cubic spline analysis

The prolonged-course group received corticosteroids for an average of 19 (14–28) days compared to nine (7–10) days in the short-course group (p < 0.001). The maximum methylprednisolone-equivalent dose of steroids was higher in the prolonged-course group (56 [36–96] vs. 32 [32–36], p < 0.001). Overall in-hospital mortality was 35.9% (n = 552). Prolonged-course corticosteroids were associated with a higher risk of death (relative risk [RR] 1.52), 95% confidence interval [95% CI] 1.24–1.89; p < 0.001. Patients in the prolonged-course group had longer hospital length of stay, higher rates of hospital infection demonstrated by greater need for antibiotics, more days under antibiotics treatment, more positive blood cultures, higher rates of ICU admission, longer ICU length of stay, and longer time spent on MV (Table 2). Table 3 shows the association of prolonged corticosteroids use and primary and secondary outcomes using the IPTW. Figure 2 (panel A) shows the non-linear association between duration of corticosteroids use and mortality in hospitalized patients using restricted cubic splines. Compared to short-course corticosteroids (short use, RR = 1), the cubic spline analysis showed an inverted U-shaped curve for mortality, with the highest risk associated with prolonged use at 30 days (RR = 1.50, 95% CI 1.21–1.78). Each additional day beyond the recommended 10 days was associated with a non-linear increase in risk.

**Table 2:** Outcomes of hospitalized patients with COVID-19 pneumonia

Outcomes	Overall (n = 1539)	Short corticosteroids (n = 412)	Prolonged corticosteroids (n = 1127)	p
All hospitalized patients (n = 1539)				
In-hospital mortality (n, %)	552 (35.9)	107 (26)	445 (39.5)	< 0.001
ICU mortality (n, %)	473 (30.7)	75 (18.2)	398 (35.3)	< 0.001
Hospital LOS (days)	23 (16–33)	17 (13–24)	25 (17–36)	< 0.001
Transfer to another facility (n, %)	138 (9)	35 (8.5)	103 (9.1)	0.771
ICU LOS (days)	16 (8–27)	11 (5–17)	19 (10–30)	< 0.001
Time spent on MV (days)	16 (10–27)	11 (6–15)	18 (11–29)	< 0.001
Number of antibiotics used	4 (2–6)	2 (1–4)	4 (2–6)	< 0.001
Days on antibiotics	16 (8–31)	11 (6–20)	20 (10–34)	< 0.001
Number of positive hemoculture	0.00 [0.00, 2.00]	0.00 [0.00, 1.00]	1.00 [0.00, 2.00]	< 0.001

	Overall (n = 622)	Short corticosteroids (n = 163)	Prolonged corticosteroids (n = 459)	p
Critically ill patients (n = 622)				
In-hospital mortality (n, %)	267 (42.9)	44 (27)	223 (48.6)	< 0.001
ICU mortality (n, %)	258 (41.5)	41 (25.2)	217 (47.3)	< 0.001
Hospital LOS (days)	25 (17–35)	18 (14–28)	27 (19–38)	< 0.001
ICU LOS (days)	21 (14–31)	14 (11–21)	23 (16–34)	< 0.001
Time spent on MV (days)	18 (12–29)	13 (7–19)	21 (14–32)	< 0.001
Number of antibiotics used	5 (3–7)	4 (2–5)	5 (4–7)	< 0.001
Days on antibiotics	23 (12–38)	13 (7–24)	26 (14–42)	< 0.001
Number of positive hemoculture	1.00 [0.00, 2.00]	1.00 [0.00, 2.00]	1.00 [0.00, 2.00]	< 0.001

Values are mean ± SD or median and interquartile range

*ICU* intensive care unit, *LOS* length of stay, *MV* mechanical ventilation

**Table 3:** Association of prolonged use of corticosteroids and outcomes using the inverse probability of treatment weighting analysis

Outcome	Relative risk	95% CI	p value
All hospitalized patients (n = 1539)			
In-hospital mortality	1.76	1.41–2.22	< 0.001
Number of antibiotics used	1.12	1.06–1.19	< 0.001
Number of positive hemocultures	1.25	1.12–1.40	< 0.001
Critically ill patients (n = 622)			
In-hospital mortality	2.37	1.67–3.47	< 0.001
Number of antibiotics used	1.19	1.09–1.29	< 0.001
Number of positive hemocultures	1.11	0.96–1.28	0.155

**Fig. 3:**
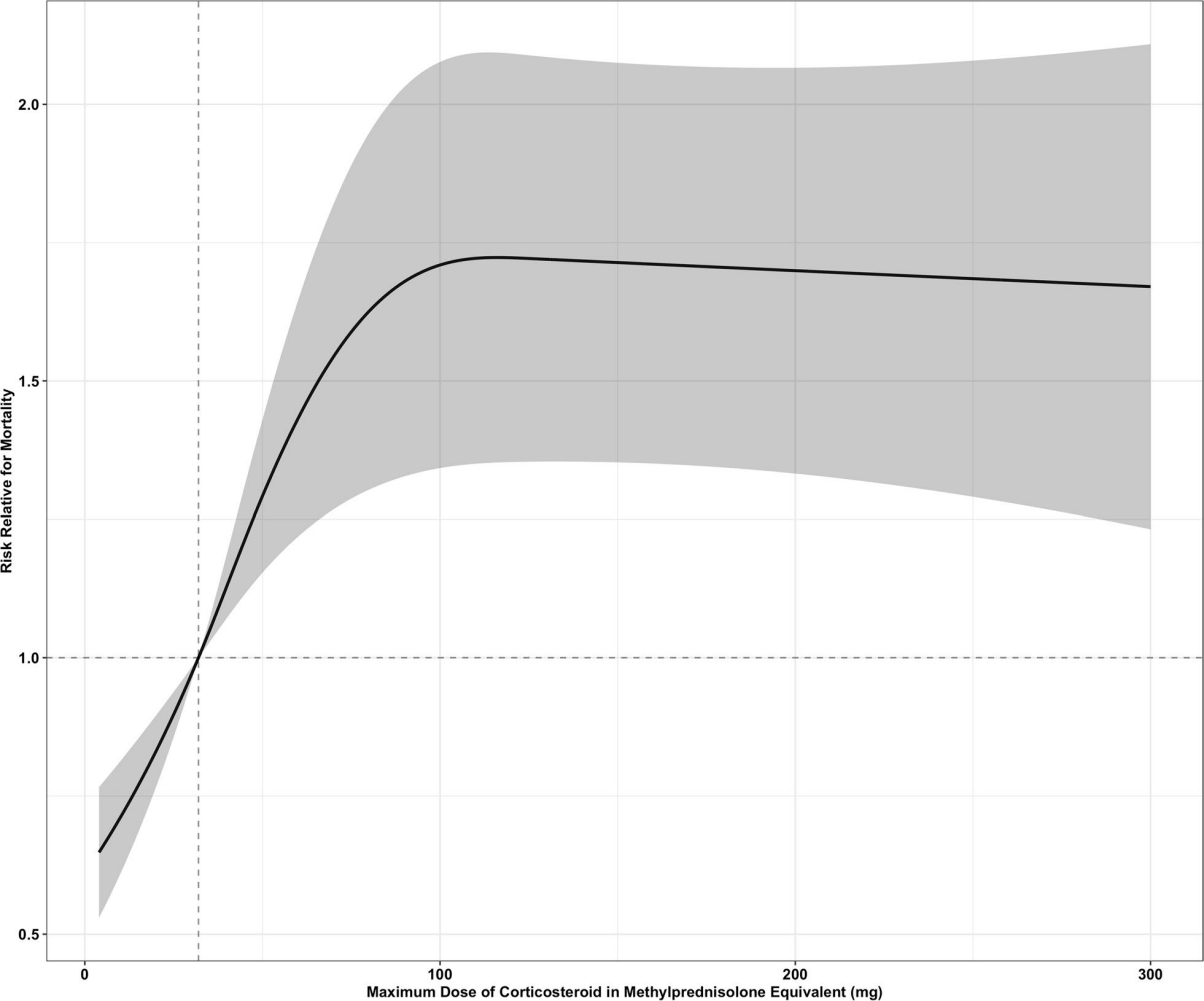
Non-linear association between dose of corticosteroids use and mortality. Non-linear association between maximum dose of corticosteroids in methylprednisolone equivalent and mortality in hospitalized patients using restricted cubic spline analysis

The spline analysis showed that as corticosteroid doses increased, mortality also presented a progressive non-linear increase until reaching a plateau at a 100 mg of methylprednisolone-equivalent dose (RR = 1.71, 95% CI 1.35–2.09) (Fig. 3). This outcome was compared to a baseline of 32 mg of methylprednisolone equivalent dose (RR = 1).

### In-hospital mortality and clinical outcomes in mechanically ventilated critically ill patients

To better understand the effect of prolonged use of corticosteroids during critical illness, we analyzed the ICU patients separately (n = 622). Table 1 shows the main characteristics of this population. The maximum methylprednisolone-equivalent dose of steroids was higher in the prolonged group (64 [40–100] vs. 32 [32–40], p < 0.001). Figure 2 (panel B) shows the non-linear association between duration of corticosteroids use and mortality in critically ill patients using restricted cubic splines.

With the IPTW model, prolonged use of corticosteroids in critically ill patients was associated with higher risk of in-hospital death (RR 2.37, 95% CI 1.67–3.47; p < 0.001), higher risk of ICU death (RR = 2.94, 95% CI 2.0–4.51; p < 0.001), and greater need for antibiotics in number and duration (RR = 1.19, 95% CI 1.10–1.29; p < 0.001) (Table 3).

### Sensitivity analyses

We performed sensitivity analyses changing the covariates in the IPTW model for in-hospital mortality and other outcomes (Additional file 1: Table S1). All models showed higher risk of mortality and infection associated with prolonged corticosteroid use. In a multivariate Poisson regression, mortality was associated with a prolonged corticosteroid use (RR = 1.60, 95% CI 1.30–1.99), age (RR = 1.02, 95% CI 1.02–1.03), but not with ICU < 48 h (RR = 1.11, 95% CI 0.90–1.37) or sex (RR = 0.89, 95% CI 0.75–1.06).

## Discussion

In this retrospective cohort study of hospitalized patients with positive SARS-CoV-2 testing, we report that the prolonged use of corticosteroids (more than 10 days) was associated with a higher risk of in-hospital death compared to 10 days of treatment. Besides, the prolonged use of corticosteroids increased hospital infections and the need for antibiotics in number and duration.

In this sample of hospitalized patients for more than 10 days, the incidence of prolonged corticosteroids use was higher than we expected (73%). The length of corticosteroids use was determined by the attending physician, assuming it would benefit in the treatment of COVID-19 pneumonia complicated by organizing pneumonia [12]. In the LUNG SAFE study, high-dose of corticosteroids was among the most common adjunctive therapy in patients with ARDS, especially after the second day of disease [13]. However, we showed in this study that the prolonged use of corticosteroids was associated with higher mortality in both for critically ill and non-critically ill patients.

The prolonged use of corticosteroids was associated with all the evaluated outcomes, which reinforces the robustness of our results. In Fig. 2A, we showed that even a day beyond the recommended 10 days was associated with increased risk. However, as we do not have data on steroid-responsive comorbidities, it is possible that some patients who received corticosteroids longer than 30 days might have a chronic condition to justify its use. Patients requiring prolonged use of corticosteroids probably had a more severe disease and could have experienced worse outcomes from it, not necessarily as a direct consequence of prolonged-course corticosteroid treatment. Besides, the protective effect of using corticosteroids for less than 10 days might simply be a marker of a milder disease severity. Thus, causal inference cannot be established from this retrospective study.

Our results showed a non-linear association between the corticosteroids dose and mortality. The analysis of splines revealed an incremental risk of mortality from 32 to 100 mg of methylprednisolone-equivalent dose, in which a plateau was reached. These findings agree with the results of the recently published work from the RECOVERY group, in which hospitalized patients with hypoxemia were randomized to receive either higher corticosteroids doses (dexamethasone 20 mg) or usual dose (dexamethasone 6 mg). They demonstrated that higher doses increased the risk of mortality [14]. Decades ago, higher doses of corticosteroids have already been associated with higher mortality in patients with ARDS [15] and in patients with septic shock [16], mainly attributed to the increased risk of infections. Taken together, these results suggest against the use of methylprednisolone-equivalent doses higher than 32 mg in hospitalized patients with COVID-19 presenting hypoxemia.

The strengths of this study are the large sample size, including non-critically and critically ill patients, and the robust statistical analysis. To the best of our knowledge, it is the best available data of the ideal length of corticosteroids for patients with COVID-19 pneumonia and certainly adds to the current knowledge of the management COVID-19 patients. However, some limitations should be addressed. First, this was a retrospective cohort study and it was not possible to establish causal inference. Although we considered all the available confounding factors, our model might still be biased, mostly due to unmeasured confounding factors. Second, not all information, especially those related to disease severity scores, could be collected in retrospect, resulting in few available variables for adjustment. Third, we included patients with positive Sars-CoV-2 testing and possibly a minority of patients did not have COVID-19. However, during the pandemic peak most patients admitted having respiratory insufficiency to our hospital. Fourth, the calculated sample size was not reached, but significant statistical differences were found regarding all outcomes. Finally, 933 patients with a hospitalization period of less than 10 days were excluded from the study and the harmful effects of corticosteroids might have been underestimated by excluding these patients.

## Conclusions

In conclusion, prolonged courses of corticosteroids were associated with risk of hospital death compared to short courses. Based on our results, we suggest against prolonged use of corticosteroids to hospitalized patients with COVID-19 until the results of randomized controlled trials add information to this current gap of knowledge.

## Supplementary Information

**Additional file 1**. Sensitivity analyses changing the covariates in the IPTW model for in-hospital mortality and infection.

## Data Availability

All available data are published in the current manuscript. Patient-level data that underlie the results reported in this article will be shared after de-identification (text, tables, and figures) to researchers who provide a methodologically sound proposal for scientific research (with approval from an internal commission). Proposals should be directed to maviana@hcpa.edu.br. A signed data access agreement will also be required.

## Abbreviations

ARDS: Acute respiratory distress syndrome
MV: Mechanical ventilation
CRP: C-reactive protein
LDH: Lactate dehydrogenase
ICU: Intensive care unit
IQR: Interquartile range
IPTW: Inverse probability of treatment weighting
RR: Relative risk
CI: Confidence interval
