# Supplementary material for: Association between prolonged corticosteroids use in COVID-19 and increased mortality in hospitalized patients: a retrospective study with inverse probability of treatment weighting analysis
Source: Crit Care. 2023 Apr 15;27:143. doi: 10.1186/s13054-023-04434-5 (PMC10105528; doi:10.1186/s13054-023-04434-5)
Supplement: Supplementary file 1 — Additional file 1. Sensitivity analyses changing the covariates in the IPTW model for in-hospital mortality and infection. [file 13054_2023_4434_MOESM1_ESM.docx]

**Supplmental Table 1.** Sensitivity analyses changing the covariates in the IPTW model

|  | RR | 2.5% | 97.5% |
| --- | --- | --- | --- |
| **Age, need for ICU admission before 48 hours, sex, hospital length of stay and CRP levels** | | | |
| Mortality | 1.76 | 1.41 | 2.22 |
| Number of antibiotics used | 1.12 | 1.06 | 1.91 |
| Number of positive hemoculture | 1.70 | 1.50 | 1.94 |
|  |  |  |  |
| **Age, sex and CRP levels** | | | |
| Mortality | 1.55 | 1.25 | 1.92 |
| Number of antibiotics used | 1.58 | 1.48 | 1.69 |
| Number of positive hemoculture | 1.77 | 1.56 | 2.01 |
| **Age, sex, CRP levels, need for ICU admission before 48 hours and need for MV** | | | |
| Mortality | 1.47 | 1.20 | 1.82 |
| Number of antibiotics used | 1.60 | 1.50 | 1.71 |
| Number of positive hemoculture | 1.90 | 1.67 | 2.17 |
| ICU: intensive care unit; MV: mechanical ventilation; CRP: C-reactive protein; IPTW: inverse probability of treatment weighting analysis. | | | |
